# Gallstone Status Classification Using Adaptive Performance-Diversity Hybrid Ensemble with Pareto-Based Multi-Objective Selection (APDHE-Pareto)

**DOI:** 10.3390/biomedicines14102139

**Published:** 2026-09-22

**Authors:** Irem Senyer Yapici

**Affiliations:** Department of Computer Engineering, Zonguldak Bulent Ecevit University, Zonguldak 67100, Türkiye; senyerirem@beun.edu.tr

**Keywords:** gallstone disease, machine learning, ensemble learning, Pareto optimization, multi-objective optimization, prediction diversity, model stability, explainable artificial intelligence, nested cross-validation

## Abstract

**Background/Objectives:** Machine-learning models may support gallstone status classification using heterogeneous clinical and body-composition variables; however, ensemble methods often rely on predefined model combinations or arbitrarily weighted performance criteria. This study proposes the Adaptive Performance-Diversity Hybrid Ensemble with Pareto-Based Multi-Objective Selection (APDHE-Pareto), a leakage-controlled multi-objective ensemble framework that integrates complementary performance measures, prediction diversity, and cross-validation stability. **Methods:** An open-access dataset comprising 319 observations and 38 predictors was divided into a development set (*n* = 255) and a held-out internal test set (*n* = 64). Candidate classifiers were evaluated using Pareto non-dominated sorting across ROC-AUC, balanced accuracy, F1-score, and Matthews correlation coefficient. Hyperparameter optimization, probability calibration, ensemble construction, and threshold optimization were performed exclusively within the development process. Performance was assessed using repeated 10-fold nested stratified cross-validation (100 outer evaluations) and a one-time evaluation on the held-out internal test set. **Results:** Among the primary model comparisons, APDHE-Pareto achieved the highest mean ROC-AUC during repeated nested cross-validation (0.859). On the held-out internal test set, it achieved the highest ROC-AUC (0.910), balanced accuracy (0.828), F1-score (0.814), Matthews correlation coefficient (0.664), and the lowest Brier score (0.131) among the evaluated models. Formal paired comparison with the prespecified Extra Trees baseline showed no statistically significant difference in classification errors or ROC-AUC. **Conclusions:** APDHE-Pareto provides a systematic and leakage-controlled framework for multi-objective ensemble selection while achieving competitive predictive performance. Independent external validation is required to confirm its generalizability and potential clinical utility.

## 1. Introduction

Gallstone disease (GD) is characterized by the formation of solid deposits within the gallbladder or biliary tract and is among the most common gastrointestinal disorders encountered in clinical practice [1,2,3]. Although gallstones remain asymptomatic in many individuals, symptomatic disease can lead to biliary colic and clinically important complications, including acute cholecystitis, cholangitis, and pancreatitis, thereby contributing to healthcare utilization and disease burden [1,4]. The development of GD is multifactorial and has been associated with sex, age, obesity, rapid changes in body weight, diabetes mellitus, metabolic syndrome, and genetic susceptibility [5,6,7]. From a pathophysiological perspective, alterations in bile composition, cholesterol supersaturation, impaired gallbladder motility, and prolonged bile retention may contribute to stone formation [8,9,10,11]. The combined influence of demographic, metabolic, biochemical, and body-composition-related factors therefore supports evaluating GD within a multivariable rather than a single-factor framework.

Conventional assessment of gallstones relies predominantly on imaging modalities, particularly abdominal ultrasonography, computed tomography (CT), and magnetic resonance cholangiopancreatography (MRCP) [12,13]. These modalities play established but different roles in clinical practice and are subject to modality-specific limitations. For example, the diagnostic quality of ultrasonography may be affected by obesity and overlying intestinal gas, whereas CT involves ionizing radiation and MRCP may be constrained by availability, cost, and the requirement for specialized interpretation [12,13,14]. These considerations have motivated interest in complementary analytical approaches that can exploit routinely collected clinical, biochemical, anthropometric, and body-composition measurements. Such approaches are not intended to replace established imaging procedures but may help determine whether routinely available multidimensional information contains discriminative patterns associated with existing gallstone status.

Artificial intelligence (AI) and machine learning (ML) have increasingly been investigated for medical classification and clinical decision-support tasks because of their ability to model complex and potentially nonlinear relationships among heterogeneous variables. In the context of GD, existing studies can broadly be grouped into imaging-based deep learning approaches, clinical-data-based ML models, and multimodal strategies combining imaging with clinical or biochemical information [15]. This growing literature suggests that both imaging and structured clinical data contain useful information for computational gallstone assessment.

A substantial proportion of previous work has focused on imaging-based deep learning. Pang et al. [16] proposed a YOLOv3-based approach for gallstone classification and cholecystitis detection from CT images and reported an overall accuracy of 86.50%. Sun et al. [17] subsequently applied a three-dimensional convolutional neural network to MRCP data in a multicenter setting and reported an area under the curve (AUC) of 0.974. Luo et al. [18] investigated YOLOv5 variants for MRCP-based analysis and reported an overall accuracy of 90.5%. Other studies have increasingly incorporated explainable AI or attention-based architectures. Kumar et al. [19], for example, incorporated local explainability into ultrasound-based analysis, whereas Chang et al. [20] investigated an attention-based approach intended to reduce dependence on highly detailed image annotations. Collectively, these studies demonstrate the potential of AI-assisted imaging for gallstone-related assessment.

Despite their promising performance, imaging-based approaches depend on the availability and quality of the underlying imaging modality. This has encouraged investigation of ML models based on structured clinical and laboratory data. Salem et al. [21] combined clinical and metabolomic information and reported an accuracy of 83%, suggesting that heterogeneous non-imaging information may provide useful predictive value. Esen et al. [22] investigated bioimpedance and laboratory measurements and reported that Gradient Boosting achieved the highest accuracy of 85.42%, while variables including vitamin D, C-reactive protein, lean mass, and total body water contributed relevant predictive information. Steinway et al. [23] also investigated ML-based identification of patients with gallstones, whereas Blum et al. [24] explored the use of laboratory and clinical variables to support decisions concerning MRCP utilization. These studies indicate that structured clinical data can provide a useful complementary source of information for gallstone-related classification.

More recent studies have applied increasingly sophisticated optimization and ensemble strategies to publicly available gallstone data. Sarker et al. [8] combined a Random Forest (RF) classifier with Sand Cat Swarm Optimization and incorporated explainability techniques such as SHapley Additive exPlanations (SHAP) and DiCE. Das et al. [25] subsequently proposed an explainable Categorical Boosting (CatBoost) framework optimized using the Sea Lion Optimization Algorithm and incorporated SHAP, Local Interpretable Model-agnostic Explanations (LIME), and DiCE for model interpretation. Li et al. [26] investigated a hybrid framework integrating feature selection, dimensionality reduction, and stacking-based ensemble learning. More recently, Akben and Yumrutaş [27] evaluated an interpretable stacking framework using the same publicly available gallstone dataset comprising 319 participants and 38 features. These studies demonstrate the increasing methodological sophistication of ML-based gallstone classification and highlight the potential benefits of optimization, ensemble learning, and explainable AI.

Beyond gallstone-specific applications, recent biomedical ensemble frameworks have also combined feature selection, prediction-probability fusion, and meta-learning. For example, Weng et al. [28] proposed a two-stage framework in which selected features and base-model probabilities were integrated within a meta-ensemble architecture. Other recent studies have investigated multi-objective feature selection, diversity-guided stacking, and multi-objective hyperparameter optimization [29,30,31]. These approaches illustrate that ensemble learning, probability fusion, prediction diversity, and multi-objective optimization have already been considered in different forms. The relevant distinction therefore concerns how these components are incorporated into the model-selection and ensemble-construction process.

Nevertheless, several methodological issues remain relevant. Many ML studies ultimately identify or optimize a single best-performing classifier, while ensemble methods may employ predefined model combinations or optimize performance according to a single criterion. However, classification quality is inherently multidimensional [32,33]. The area under the receiver operating characteristic curve (ROC-AUC), balanced accuracy, F1-score, and Matthews correlation coefficient (MCC), for example, capture different aspects of predictive behavior [34]. Reducing these criteria to a single weighted score requires specification of numerical weights, which may be difficult to justify when no explicit clinical utility function is available. Moreover, selecting ensemble constituents solely according to predictive performance may overlook whether candidate classifiers provide complementary predictions or whether their performance remains stable across different data partitions [35,36].

Multi-objective optimization provides a natural framework for addressing this problem because several performance criteria can be considered simultaneously without requiring their reduction to a single weighted objective. Pareto concepts have previously been investigated in classifier and ensemble selection, including approaches that consider predictive performance and diversity [37,38,39,40]. Accordingly, the methodological contribution of the present study is not the introduction of Pareto optimization, ensemble learning, probability calibration, or probability-level fusion individually. In Adaptive Performance-Diversity Hybrid Ensemble with Pareto-Based Multi-Objective Selection (APDHE-Pareto), the multi-objective formulation is instead applied directly to constituent-model selection. Candidate classifiers are first evaluated through Pareto non-dominated sorting using ROC-AUC, balanced accuracy, F1-score, and MCC as simultaneous performance objectives. Prediction diversity and cross-validation stability are subsequently used as hierarchical selection characteristics rather than as additional weighted objectives. Although this procedure avoids numerical scalarization of the primary performance objectives, the subsequent lexicographic ranking applies a prespecified hierarchical preference among the selection characteristics and should therefore not be interpreted as a preference-free selection procedure. Ensemble subset size and the final decision threshold are then determined from training-only out-of-fold predictions. Thus, the methodological distinction of APDHE-Pareto lies primarily in the organization of the constituent-selection process and its leakage-controlled implementation.

Accordingly, this study proposes APDHE-Pareto for gallstone status classification. Rather than identifying a single best-performing classifier or collapsing multiple evaluation criteria into a predefined weighted score, APDHE-Pareto treats AUC, balanced accuracy, F1-score, and MCC as simultaneous objectives through Pareto non-dominated sorting. Prediction diversity and cross-validation stability are subsequently incorporated as complementary selection characteristics to distinguish candidate classifiers with different predictive behaviors and performance consistency across validation partitions. Importantly, these components are used to inform ensemble construction rather than under the assumption that each criterion will independently or monotonically improve predictive performance.

The selected calibrated classifiers are combined at the probability level using uniform averaging. Hyperparameter optimization, probability calibration, constituent-model selection, ensemble-size determination, and decision-threshold optimization are performed using training data only. The complete modeling procedure is evaluated using repeated nested cross-validation and a separate held-out internal test set, thereby separating data-dependent model development from final performance assessment and reducing the risk of optimistic bias. The main contributions of this work are summarized as follows:

1. Pareto-based constituent selection without numerical scalarization of the primary performance objectives: Candidate classifiers are evaluated simultaneously using ROC-AUC, balanced accuracy, F1-score, and MCC through Pareto non-dominated sorting rather than being collapsed into a single weighted composite score. The subsequent lexicographic ranking applies a prespecified hierarchical preference and therefore should not be interpreted as a preference-free selection procedure.
2. Hierarchical incorporation of prediction diversity and stability: Prediction diversity and cross-validation stability are used to refine constituent ordering after multi-objective performance assessment rather than being introduced as additional weighted objectives. This design separates primary performance-based selection from complementary information concerning prediction redundancy and validation consistency.
3. Leakage-controlled end-to-end ensemble development: Hyperparameter optimization, probability calibration, constituent-model ranking and selection, ensemble-size determination, and decision-threshold optimization are restricted to the corresponding training data and derived from nested out-of-fold predictions, preventing validation or held-out test information from influencing data-dependent model-development decisions.
4. Rigorous evaluation of the complete adaptive procedure: Rather than evaluating only a fixed final ensemble, the complete APDHE-Pareto development procedure is repeated within a 10-fold nested stratified cross-validation design repeated 10 times, followed by a one-time evaluation on an untouched held-out internal test set.
5. Evaluation against heterogeneous individual classifiers and stronger ensemble baselines: APDHE-Pareto is evaluated against individually optimized classifiers representing different learning paradigms and against complementary ensemble strategies, including uniform soft voting, top-k averaging, greedy forward ensemble selection, and logistic stacking.
6. Component-level and prediction-level analysis: Ablation experiments are used to quantify the incremental contributions of the performance, diversity, and stability components rather than assuming that each component necessarily improves predictive performance. In particular, the ablation analysis explicitly examines whether the diversity and stability components provide incremental value within the proposed framework, and the results are interpreted conditionally rather than as evidence of monotonic improvement. Post hoc SHAP and LIME analyses are additionally used to characterize global and local attribution patterns underlying the final model predictions.

The primary objective of this study is to develop and evaluate APDHE-Pareto for gallstone status classification using clinical, biochemical, anthropometric, and body-composition variables available in the study dataset. Specifically, we investigate whether these heterogeneous predictors can be integrated within a leakage-controlled multi-objective ensemble framework to discriminate between individuals with and without gallstones. In addition to comparing APDHE-Pareto with individually optimized ML classifiers, we examine the incremental roles of multi-metric performance assessment, prediction diversity, and cross-validation stability in adaptive ensemble construction. Accordingly, the study is intended not only to evaluate predictive performance in the present gallstone dataset but also to examine the methodological behavior of an adaptive multi-objective ensemble-selection strategy under repeated internal validation. The present study should therefore be regarded primarily as a methodological evaluation of the APDHE-Pareto framework rather than as validation of a screening, triage, or standalone diagnostic tool. Any potential future role as a complementary clinical decision-support approach would require prospective evaluation and independent external validation.

## 2. Materials and Methods

This study was designed to develop and evaluate a ML framework for gallstone status classification using clinical, biochemical, anthropometric, and body-composition variables. A publicly available gallstone dataset was analyzed using a leakage-controlled experimental design that strictly separated model development from final performance evaluation. Multiple conventional and ensemble ML classifiers were systematically evaluated, and the proposed APDHE-Pareto framework was developed to integrate multi-objective predictive performance, prediction diversity, and cross-validation stability through Pareto-based model selection without numerically scalarizing the primary performance objectives into a single weighted composite score. Model development was conducted within a nested cross-validation framework, followed by a one-time evaluation on a held-out internal test set. The study design and analytical workflow are illustrated in Figure 1. The following subsections describe the dataset, study design, data preprocessing, candidate classifiers, hyperparameter optimization, probability calibration, APDHE-Pareto framework, validation and performance evaluation, statistical analysis, ablation analysis, and post hoc explainability procedures.

### 2.1. Dataset

This study utilized the open-access Gallstone Dataset available from the UCI Machine Learning Repository [22]. The data were originally collected at Ankara VM Medical Park Hospital between June 2022 and June 2023 with the approval of the Ankara City Hospital Ethics Committee. In the original study, informed consent was obtained from all participants in accordance with the Declaration of Helsinki. Individuals who had previously undergone gallbladder surgery were excluded from the study.

The dataset comprised 319 participants, including 161 individuals with gallstones and 158 controls without gallstones. A total of 38 predictor variables were available, encompassing demographic and clinical characteristics, body-composition measurements, and biochemical parameters. Body-composition measurements were obtained using the Tanita MC-780 device (TANITA Corporation, Tokyo, Japan), while liver-related assessments were performed using ultrasonography. Accordingly, some predictor information was derived from ultrasonographic assessment rather than exclusively from pre-imaging clinical or laboratory measurements. The publicly available dataset documentation does not provide sufficient information to establish the precise temporal ordering of these ultrasound-derived assessments relative to the determination of gallstone status. Therefore, their availability before outcome determination cannot be assumed, and the present model should not be interpreted as a pre-ultrasound screening tool. Accordingly, the dataset represents a heterogeneous combination of clinical, biochemical, anthropometric, body-composition, and ultrasound-derived information rather than a purely laboratory-based dataset. The main characteristics of the study population according to gallstone status are summarized in Table 1.

### 2.2. Study Design

The dataset was stratified into a development set comprising 80% of the observations (*n* = 255) and a held-out internal test set comprising the remaining 20% (*n* = 64), while preserving the distribution of gallstone status. The development set was used exclusively for all data-dependent model-development procedures, whereas the held-out internal test set remained isolated throughout model development. Within the development set, model development and internal validation were conducted using a nested stratified cross-validation strategy. Hyperparameter optimization, probability calibration, constituent-model selection, APDHE-Pareto ensemble construction, and decision-threshold determination were performed exclusively within the corresponding training partitions. The complete modeling pipeline was evaluated using repeated nested cross-validation, whereas the held-out internal test set was accessed only once after all development decisions had been finalized. This design was adopted to minimize information leakage and reduce optimistic bias in the estimation of generalization performance.

### 2.3. Data Preprocessing

Prior to model development, the dataset was examined for data completeness, variable types, and target-variable encoding. No missing observations were identified; therefore, no missing-data imputation was required. Gallstone status was defined as the binary outcome. In the original dataset, a value of 0 represented the presence of gallstones, whereas a value of 1 represented their absence. Accordingly, class 1 (absence of gallstones) was treated as the positive class throughout model development, probability estimation, and performance evaluation. Consequently, predicted probabilities represent the probability of gallstone absence. Sensitivity and precision therefore quantify the correct identification of individuals without gallstones, whereas specificity quantifies the correct identification of individuals with gallstones.

Predictor variables were separated from the target variable before model development. Preprocessing was implemented within model-specific pipelines to prevent information leakage. For algorithms requiring feature scaling, standardization was performed using parameters estimated exclusively from the corresponding training partition, and the fitted transformation was subsequently applied to the associated validation or test observations without re-estimation. Algorithms that did not require feature scaling were fitted directly using the original predictor values within the corresponding training partitions. Preprocessing operations were therefore contained within the relevant training-validation boundaries throughout hyperparameter optimization and nested cross-validation. This pipeline-based implementation ensured that preprocessing parameters were estimated exclusively from the corresponding training data and subsequently applied to validation or held-out test observations without re-estimation, thereby preventing information leakage and reducing the risk of optimistically biased performance estimates.

To facilitate clinically intuitive interpretation, a secondary disease-oriented analysis was additionally performed in which gallstone presence was treated as the positive class. Disease-oriented sensitivity, specificity, precision, and F1-score were recalculated from the same frozen predictions by reversing the class orientation. This analysis did not involve model refitting, threshold re-optimization, or modification of the original model-selection procedure.

### 2.4. Candidate ML Algorithms

A heterogeneous set of ML classifiers was evaluated to represent diverse learning paradigms and decision structures for gallstone status classification [41]. The candidate model pool comprised Logistic Regression (LR), Support Vector Machine (SVM), RF, Extra Trees (ET), Gradient Boosting (GB), AdaBoost, Light Gradient Boosting Machine (LightGBM), and CatBoost. LR was included as a conventional linear probabilistic classifier, whereas SVM represented a margin-based learning approach [42]. Tree-based ensemble methods included RF and ET, which employ randomized tree construction, together with the boosting algorithms GB and AdaBoost. LightGBM and CatBoost were additionally included as modern gradient-boosting implementations capable of modeling nonlinear relationships and complex interactions among predictor variables [43,44,45].

This heterogeneous candidate pool was selected to provide complementary predictive behaviors across linear, margin-based, bagging-based, randomized-tree, and boosting-based learning paradigms. Such methodological diversity was particularly relevant to APDHE-Pareto, whose constituent-model selection considers predictive performance, prediction diversity, and cross-validation stability during ensemble construction [46,47]. Hyperparameter optimization was performed independently for each candidate classifier, as described in the following section.

#### Strong Ensemble Baselines

To provide a more stringent methodological benchmark for APDHE-Pareto, four additional ensemble strategies were evaluated: uniform soft voting across all candidate classifiers, top-k averaging based on individual OOF ROC-AUC ranking, greedy forward ensemble selection, and logistic stacking. All ensemble-construction and selection decisions were based exclusively on training-derived out-of-fold predictions within the corresponding development partitions. For top-k averaging, candidate classifiers were ranked according to their individual OOF ROC-AUC, and the ensemble size was selected using the prespecified OOF criteria. Greedy ensemble selection was implemented as forward selection without replacement, sequentially adding the candidate that optimized OOF ROC-AUC, followed by Brier score and balanced accuracy as deterministic tie-breaking criteria. Logistic stacking used the candidate-model OOF probabilities as meta-features and logistic regression as the meta-learner, with cross-validated meta-level predictions generated within the training data. Uniform soft voting assigned equal weight to the calibrated probabilities of all candidate classifiers. For all four ensemble baselines, the decision threshold was determined from training-only OOF predictions using Youden’s J and was subsequently frozen before evaluation on the corresponding outer-validation or held-out internal test data. Thus, the held-out internal test set was not used for constituent selection, ensemble construction, threshold optimization, or other model-development decisions.

### 2.5. Hyperparameter Optimization

Hyperparameter optimization was performed exclusively within the training partitions of the nested cross-validation procedure using randomized search. For each candidate classifier, a model-specific hyperparameter search space was predefined and explored using stratified three-fold cross-validation. Up to six randomly sampled hyperparameter combinations were evaluated for each classifier, with ROC-AUC used as the optimization criterion. The search budget was intentionally limited to a maximum of six randomly sampled configurations per classifier to ensure a consistent level of computational effort across the heterogeneous candidate-model pool. Because hyperparameter optimization was repeated independently within each outer-training partition of the repeated nested cross-validation procedure and was followed by probability calibration, out-of-fold prediction generation, and ensemble construction, substantially larger search budgets would have considerably increased the computational burden of the complete analysis. The same maximum search budget was therefore applied across candidate classifiers as a pragmatic compromise between model optimization and computational feasibility. The hyperparameter configuration yielding the highest mean cross-validated ROC-AUC was subsequently refitted using the corresponding training partition before probability calibration and further model-development procedures. No information from the outer validation folds or the held-out internal test set was used during hyperparameter optimization. The predefined hyperparameter search spaces are summarized in Table 2.

### 2.6. Probability Calibration

Because APDHE-Pareto combines calibrated class probabilities rather than hard class assignments, probability calibration was incorporated into the model-development pipeline. Following hyperparameter optimization, each candidate classifier was calibrated using Platt scaling (sigmoid calibration) implemented with CalibratedClassifierCV. Calibration was performed using stratified three-fold internal cross-validation within the corresponding training partition, with shuffling enabled and a deterministic random seed derived from the global seed. The cross-validated ensemble form of calibration was used. No observations from the corresponding outer-validation fold or the held-out internal test set were used to fit the calibration procedure, thereby preventing information leakage [48,49]. For a candidate classifier $M_{i}$, the calibrated probability for an observation x was expressed as:

(1) $$p_{i}(x)=P(Y=1\;|\;x,\;M_{i})$$

In this equation, $p_{i}\;(x)$ denotes the calibrated probability of class 1 (absence of gallstones) for observation x, as estimated by candidate classifier $M_{i}$. The calibrated probabilities were subsequently used to generate out-of-fold predictions for constituent-model selection, ensemble construction, and final probability estimation [49].

Probabilistic performance was evaluated using the Brier score, whereas calibration was assessed separately using calibration curves, calibration intercept, and calibration slope. For the held-out internal test evaluation, calibration curves were constructed by comparing observed outcome frequencies with mean predicted probabilities across five quantile-based probability groups. Calibration intercept and slope were estimated by fitting a logistic recalibration model to the logit-transformed predicted probabilities, with ideal calibration corresponding to an intercept of 0 and a slope of 1. The Brier score was defined as:

(2) $$BS=\frac{1}{N}\sum_{n=1}^{N}{\left(p_{n}\;-\;y_{n}\right)}^{2}$$

where $p_{n}\;$ denotes the predicted probability of class 1 for observation n, $y_{n}\;$ denotes the corresponding observed binary outcome, and N is the total number of observations. Lower Brier scores indicate better overall probabilistic accuracy; however, the Brier score does not measure calibration in isolation because it reflects multiple aspects of probabilistic prediction. Calibration was therefore additionally assessed using calibration curves and calibration intercept and slope [50,51].

### 2.7. Proposed APDHE-Pareto Framework

APDHE-Pareto was developed to construct an ensemble by jointly considering predictive performance, prediction diversity, and cross-validation stability without assigning predefined numerical weights to the primary performance objectives. Candidate classifiers were evaluated using training-only out-of-fold (OOF) predictions generated within the nested validation procedure. These OOF predictions formed the basis for multi-objective performance assessment, diversity and stability estimation, deterministic model ranking, ensemble subset selection, probability fusion, and decision-threshold optimization. Thus, all data-dependent decisions involved in APDHE-Pareto construction were restricted to the corresponding training partitions, thereby preventing information leakage during model development [29,47]. For multi-objective performance assessment, each candidate classifier was characterized using ROC-AUC, balanced accuracy, F1-score, and Matthews correlation coefficient (MCC). The threshold-dependent performance measures, namely balanced accuracy, F1-score, and MCC, were calculated from the training-only OOF probabilities using a fixed probability threshold of 0.50 for all candidate classifiers. No model-specific threshold optimization was performed during Pareto ranking; thus, the 0.50 threshold provided a common operating point for evaluating these measures across candidate models. Because MCC ranges from −1 to 1, it was transformed to the [0, 1] interval as [52]:

*MCC*′ = (*MCC* + 1)/2 (3)

All four criteria were treated as maximization objectives and evaluated using sequential Pareto non-dominated sorting. A classifier was considered dominated if another classifier performed at least as well across all four objectives and strictly better on at least one. The non-dominated classifiers constituted the first Pareto layer, and subsequent layers were obtained recursively after removing classifiers assigned to the preceding non-dominated set. Lower Pareto-layer indices therefore represented more favorable multi-objective performance. This procedure enabled simultaneous consideration of complementary performance criteria without collapsing them into a predefined weighted composite score [53]. To encourage complementary prediction behavior among the selected constituent classifiers, prediction diversity was quantified from pairwise OOF class predictions using the disagreement measure and the Q-statistic. The Q-statistic was transformed as:

(4) QDiv = (1  −  Q) / 2

In this equation, larger transformed values represented greater predictive diversity between classifiers i and  j [54]. Pairwise combined diversity was then calculated as the arithmetic mean of the disagreement measure and the transformed Q-based diversity measure:

(5) $$D_{ij}=\frac{{Disagreement}_{ij}+{QDiv}_{ij}}{2}$$

When the Q-statistic was undefined because its denominator was zero, pairwise diversity was defined using the disagreement measure alone [55]. A model-level diversity score was subsequently obtained for each candidate classifier by averaging its pairwise combined-diversity values across all other candidate classifiers:

(6) $$D_{i}=\frac{1}{M-1}\sum_{j\neq i}D_{ij}$$

In this equation, M denotes the total number of candidate classifiers. Higher Di values therefore indicated greater average predictive diversity relative to the remaining candidate-model pool [55]. Model stability was characterized using the variability of fold-level AUC values obtained during training-only OOF prediction generation. For classifier i, stability was quantified as:

*Stability_i_* = 1/[1 + *SD*(*AUC_i_*)] (7)

In this equation, higher values indicated more consistent discrimination across validation folds [56]. Candidate classifiers were subsequently ranked using a deterministic lexicographic ranking strategy. Lower Pareto-layer indices were prioritized first, followed sequentially by higher model-level diversity, higher stability, higher AUC, higher balanced accuracy, higher F1-score, and higher transformed MCC. Alphabetical model name was used as the final deterministic tie-breaking criterion when all preceding ranking criteria were equivalent. This ordering was prespecified to maintain a separation between primary multi-objective performance assessment and secondary ensemble-construction characteristics. Pareto layer was given first priority to preserve non-dominated performance across the four primary objectives, whereas diversity and stability were subsequently used to favor prediction complementarity and consistency among similarly positioned candidates. The individual performance measures were then applied as deterministic lexicographic criteria, with ROC-AUC given precedence because discrimination was the primary performance measure of the study. Balanced accuracy, F1-score, and MCC were used as subsequent deterministic criteria. This ordering was prespecified as an operational ranking rule rather than as a clinically or universally optimal preference structure. Diversity and stability were therefore incorporated as hierarchical selection characteristics rather than as additional weighted performance objectives [57]. This design preserved the Pareto-based formulation while allowing diversity and stability to influence model selection without introducing additional optimization weights.

The procedure involves two distinct levels of preference. At the Pareto-ranking stage, the four primary performance objectives are treated simultaneously without scalarization or predefined numerical weights. At the subsequent lexicographic-ranking stage, however, a prespecified hierarchical order is applied to the selection characteristics. Thus, although the procedure avoids numerical weighting of the primary Pareto objectives, it is not preference-free, because the lexicographic ordering encodes a specific priority structure. This hierarchy represents a methodological design choice, and alternative orderings could potentially lead to different constituent-model rankings and ensemble compositions. Sensitivity to the prespecified lexicographic hierarchy was assessed on the development data using three alternative ranking orders that prioritized stability, ROC-AUC, or balanced accuracy. Ensemble selection and OOF performance were compared with the original diversity-first hierarchy, without using the held-out internal test set. Accordingly, this analysis was intended specifically to assess the sensitivity of the model-selection procedure to the prespecified lexicographic ranking hierarchy within the development data and should not be interpreted as evidence that out-of-sample generalization performance is insensitive to the ranking hierarchy.

The number of constituent classifiers was determined exclusively from training-only OOF predictions. Candidate ensemble sizes of K = 2, 3, 4, 5, and 6 were evaluated. For each candidate size K, the ensemble was defined as the first K classifiers in the lexicographically ranked model list; alternative non-prefix subsets were not evaluated. The calibrated OOF probabilities of the classifiers included in each candidate subset were combined using uniform averaging. Candidate ensemble sizes were then compared lexicographically by prioritizing higher OOF AUC, followed by lower OOF Brier score, higher OOF balanced accuracy, and, when the preceding criteria were equivalent, a smaller ensemble size. For the balanced-accuracy criterion, class predictions were obtained using a threshold determined from the corresponding fused training-only OOF probabilities. This procedure allowed the ensemble size to be selected without using information from the corresponding outer-validation or held-out test observations [51]. After subset selection, the calibrated probabilities of the selected classifiers were combined using uniform probability averaging:

(8) $$\hat{p}\_\mathrm{APDHE}(x)=1/K{\sum_{i=1}}^{K}{\hat{p}}_{i}(x)$$

where K denotes the number of selected ensemble members and p^ i (x) denotes the calibrated probability produced by the i-th selected classifier for observation x. Thus, all selected constituent models contributed equally to the final APDHE-Pareto probability estimate, avoiding both learned and manually specified ensemble weights [58]. Finally, the classification threshold was determined from the fused training OOF probabilities by maximizing Youden’s J statistic:

*J*(*t*) = *Sensitivity*(*t*) + *Specificity*(*t*) − 1 (9)

t* = *arg max_t_ J*(*t*) (10)

In the event of equivalent candidate thresholds, balanced accuracy and proximity to 0.50 were used as deterministic secondary criteria [59]. The selected threshold was fixed before evaluation on unseen outer-validation or held-out test observations. Accordingly, hyperparameter optimization, probability calibration, APDHE-Pareto ranking, ensemble subset selection, probability fusion, and threshold optimization were performed exclusively using the corresponding training data, without using outcome information from the observations on which generalization performance was subsequently evaluated. These prespecified procedures, together with the deterministic random-seed scheme described below, were used to facilitate computational reproducibility and to avoid post hoc adjustment based on validation or held-out test performance.

A global random seed of 42 was used as the base seed throughout the analysis. The development/held-out split and the repeated outer cross-validation splitter used this seed directly. Within repeated nested cross-validation, the base seed for each outer evaluation was defined as 42 + 10,000o, where o denotes the 1-based outer-evaluation index across all repeats. For nested OOF generation, the seed for candidate model M in inner fold f was calculated as 42 + 10,000o + 1000f + Σ ord(c), where f denotes the 1-based inner-fold index and ord(c) denotes the Python Unicode code point of each character in the model identifier. The model identifiers used in this calculation were LR, SVM, RF, ET, GB, ADB, LGBM, and CAT. The same derived seed was used for randomized hyperparameter optimization, whereas probability calibration used the corresponding seed plus 101. For the final candidate-model fits within each outer-training partition, the seed was defined as 42 + 10,000o + 50,000 + 1000m, where m denotes the zero-based model index in the fixed order LR, SVM, RF, ET, GB, ADB, LGBM, and CAT. For the final APDHE-Pareto fit on the complete development set, the base seed was set to 42 + 999,999, after which the fold-, model-, calibration-, and final-fitting offsets defined above were applied unchanged.

For clarity, the constituent-ranking, subset-selection, probability-fusion, and threshold-selection procedure is summarized in Algorithm 1.
**Algorithm 1.** APDHE-Pareto ensemble-selection procedure within each training partition**Input:** Training partition $D_{train}$ candidate classifier set M; allowable ensemble sizes $K=\left\{2,3,4,5,6\right\}$
**Output:** Selected ensemble $E^{*}\;$and decision threshold $t^{*}$
For each candidate classifier, generate calibrated training-OOF probability predictions using the nested model-development procedure.Using the OOF predictions, calculate ROC-AUC, balanced accuracy, F1-score, and MCC for each candidate classifier at a common probability threshold of 0.50.Transform MCC to the [0, 1] interval and assign Pareto layers by sequential non-dominated sorting across ROC-AUC, balanced accuracy, F1-score, and transformed MCC.Calculate model-level prediction diversity from pairwise disagreement and transformed Q-statistic measures.Calculate model stability as 1/[1+SD(AUC)], where SD(AUC) is obtained from the fold-level OOF AUC values.Rank candidate classifiers lexicographically by: lower Pareto-layer index, higher diversity, higher stability, higher ROC-AUC, higher balanced accuracy, higher F1-score, higher transformed MCC, and alphabetical model name as the final deterministic tie-breaker.
For each allowable ensemble size K∈{2,3,4,5,6}, define the candidate ensemble as the first K classifiers in the ranked list. Non-prefix subsets are not evaluated.For each candidate ensemble, uniformly average the calibrated OOF probabilities of its constituent classifiers.Determine an OOF decision threshold for each candidate ensemble by maximizing Youden’s J; when equivalent thresholds occur, prioritize higher balanced accuracy and then the threshold closest to 0.50.Compare candidate ensemble sizes lexicographically by higher OOF ROC-AUC, lower OOF Brier score, higher OOF balanced accuracy, and smaller ensemble size.Select the highest-ranked candidate ensemble as $E^{*}$.Recalculate the fused OOF probabilities for $E^{*}$ and determine the final training-derived threshold $t^{*}$ using the same Youden’s J rule.Fit the corresponding calibrated candidate models on the complete current training partition.Apply the fixed ensemble composition $E^{*},\;$ uniform probability averaging, and fixed threshold $t^{*}\;$ to the unseen validation or test observations.


### 2.8. Model Validation and Performance Evaluation

Model performance was evaluated using repeated nested stratified cross-validation to obtain a robust estimate of generalization performance while preserving class proportions across folds. The complete model-development procedure, including hyperparameter optimization, probability calibration, constituent-model selection, APDHE-Pareto ensemble construction, and decision-threshold optimization, was performed independently within each training partition. Performance was then assessed on the corresponding outer validation fold, ensuring that observations used for evaluation did not contribute to data-dependent model-development decisions. The nested cross-validation procedure consisted of 10 outer folds and was repeated 10 times, yielding 100 outer-fold evaluations for each modeling approach.

Discriminative performance was primarily assessed using ROC-AUC. To provide a broader characterization of classification performance, balanced accuracy, sensitivity, specificity, precision, F1-score, and MCC were additionally calculated. Probabilistic prediction performance was evaluated using the Brier score [60].

After completion of model development and repeated nested cross-validation, the finalized model configurations were refitted using the complete development set. Generalization performance was subsequently assessed once on the held-out internal test set, which had remained isolated from model development, hyperparameter optimization, probability calibration, ensemble selection, and threshold determination. The same performance measures were calculated on the held-out internal test set using identical evaluation procedures to enable direct comparison between APDHE-Pareto and the individual candidate classifiers.

The analyses were conducted using Python 3.12.3, with NumPy 1.26.4, pandas 2.3.3, scikit-learn 1.5.2, SciPy 1.15.1, LightGBM 4.5.0, CatBoost 1.2.7, and SHAP 0.47.1 and LIME 0.2.0.1. The exact software versions were recorded automatically during execution of the analysis pipeline.

### 2.9. Statistical Analysis

Performance estimates obtained from repeated nested cross-validation were summarized descriptively using the mean and standard deviation across the outer-validation evaluations. Because observations contribute to multiple training and validation partitions across repeated cross-validation, the 100 outer-fold estimates are statistically dependent and were not treated as independent observations for inferential confidence-interval estimation. For the final paired statistical comparison, the strongest individual baseline was selected exclusively from the development data as the classifier with the highest unrounded mean ROC-AUC across the repeated nested cross-validation evaluations. Thus, baseline selection was completed without reference to performance on the held-out internal test set. Formal paired statistical comparisons were subsequently performed between APDHE-Pareto and the selected baseline using paired predictions obtained from the same held-out test observations. Differences in paired hard-classification outcomes were evaluated using the two-sided exact McNemar test. Differences in discriminative performance were assessed using the paired difference in ROC-AUC between APDHE-Pareto and the selected baseline. A stratified paired bootstrap procedure with 5000 resamples was used to estimate the 95% confidence interval for the AUC difference. Bootstrap-based 95% confidence intervals were also calculated for the principal performance measures on the held-out internal test set. Receiver operating characteristic curves were constructed from the predicted probabilities obtained on the held-out internal test set. For each model displayed in the ROC analysis, 95% confidence intervals for the AUC were estimated using 5000 stratified bootstrap resamples of the held-out test observations. Accordingly, formal statistical inference was restricted to paired predictions generated for the same previously unseen held-out test observations, whereas repeated nested cross-validation results were interpreted descriptively to characterize model performance, variability, and development-stage robustness rather than for inferential hypothesis testing.

### 2.10. Ablation Analysis

An ablation analysis was conducted to examine the individual contributions of prediction diversity and stability to the proposed APDHE-Pareto framework. Four configurations were evaluated: (i) Pareto-based performance selection alone, (ii) Pareto-based selection combined with prediction diversity, (iii) Pareto-based selection combined with stability, and (iv) the complete APDHE-Pareto framework incorporating both diversity and stability.

For each configuration, model ranking, subset selection, and decision-threshold determination were performed exclusively using information derived from the corresponding outer-training partition. The remaining model-development procedures were kept identical across all ablation configurations to ensure that observed performance differences could be attributed solely to the inclusion or exclusion of the diversity and stability components. The resulting ensemble was subsequently evaluated on the unseen outer-validation fold. Validation labels were not used during constituent-model ranking or ensemble selection. Ablation performance was summarized across the repeated outer cross-validation evaluations using the same classification and probabilistic performance measures applied to the primary model comparisons. This design enabled the incremental contribution of the diversity and stability components to be examined without introducing validation-set information into the model-selection procedure.

The purpose of the ablation analysis was not to assume that each additional selection criterion would necessarily improve predictive performance, but to quantify the incremental contribution of diversity and stability within the proposed ensemble-selection framework. Because prediction diversity may trade off against individual predictive strength or stability, the full APDHE-Pareto configuration was not expected a priori to dominate every reduced configuration on every performance metric. Accordingly, the ablation results were interpreted in terms of the marginal contribution and interaction of the individual selection components rather than as evidence that increasing methodological complexity necessarily yields superior predictive performance.

### 2.11. Explainability Analysis

Post hoc explainability analyses were performed for the final APDHE-Pareto model using SHAP and LIME. These analyses were intended to characterize model attribution patterns rather than to establish causal relationships between individual predictors and gallstone status.

For global attribution analysis, the model-agnostic Kernel SHAP algorithm was applied to the final APDHE-Pareto probability predictions. Kernel SHAP was selected because APDHE-Pareto combines heterogeneous constituent classifiers at the probability level and therefore cannot be interpreted using model-specific explainability methods designed for individual tree-based models. A random sample of 60 observations from the development set was used as the SHAP background distribution, with sampling performed using a fixed random seed (random_state = 42). SHAP explanations were generated for a random sample of 60 observations from the held-out internal test set using the same fixed random seed. Global feature importance was quantified as the mean absolute SHAP value across the explained observations, with larger values indicating a greater average contribution to variation in the model output.

LIME was additionally used to provide an illustrative local explanation for the first observation in the held-out internal test set. This observation was selected according to its fixed position in the held-out dataset rather than on the basis of its prediction, outcome, or explanatory pattern. The explainer was constructed using the development data and generated a local approximation of the APDHE-Pareto prediction around the selected observation. The resulting feature contributions were used to illustrate how individual variables influenced the model output for that specific case. Both SHAP and LIME were used exclusively as post hoc model-interpretation techniques to explain prediction behavior and were not interpreted as evidence of causal, mechanistic, or clinical relationships between individual predictors and gallstone status.

## 3. Results

The proposed APDHE-Pareto framework was evaluated for the classification of gallstone status using demographic, clinical, biochemical, anthropometric, and body-composition variables. Its performance was compared with that of individually optimized ML classifiers across discrimination, classification, and probabilistic performance measures. Results from repeated nested cross-validation are presented first, followed by performance on the held-out internal test set and formal statistical comparison with the individual baseline selected exclusively from the development cross-validation results. Finally, ablation and post hoc explainability analyses are reported to examine the contribution of the APDHE-Pareto components and the variables associated with its classification decisions. The comparative performance of APDHE-Pareto and the individual candidate classifiers across the repeated nested cross-validation evaluations is summarized in Table 3.

As summarized in Table 3, APDHE-Pareto achieved the highest mean ROC-AUC (0.8585) across the repeated nested cross-validation evaluations, followed by ET (0.8514) and CatBoost (0.8496). Although APDHE-Pareto did not achieve the best value for every individual performance metric, its mean balanced accuracy (0.7755) was comparable to those of CatBoost (0.7754) and ET (0.7717). CatBoost achieved the lowest mean Brier score (0.1592), indicating the lowest overall probabilistic prediction error among the evaluated models, whereas APDHE-Pareto achieved a mean Brier score of 0.1632. Overall, these results indicate that APDHE-Pareto consistently achieved the highest average discriminative performance during repeated nested cross-validation while maintaining competitive classification and probabilistic performance relative to the strongest individual classifiers. As an additional sensitivity analysis using the expanded hyperparameter-search budget, APDHE-Pareto was benchmarked against four stronger ensemble strategies under the same repeated nested cross-validation framework. Logistic stacking achieved a mean ROC-AUC of 0.8689 ± 0.0748, uniform soft voting across all eight candidate classifiers achieved 0.8684 ± 0.0731, greedy forward ensemble selection achieved 0.8651 ± 0.0765, and top-k ROC-AUC-based averaging achieved 0.8609 ± 0.0785. Under the same expanded-search analysis, APDHE-Pareto achieved a mean ROC-AUC of 0.8613 ± 0.0792. Thus, APDHE-Pareto did not demonstrate superior discrimination over the stronger ensemble comparators in this sensitivity analysis, although its performance remained broadly comparable. In particular, logistic stacking and uniform soft voting showed modestly higher mean ROC-AUC values. The comparative performance of APDHE-Pareto and the four strong ensemble baselines in the expanded-search sensitivity analysis is summarized in Table 4. These findings indicate that the contribution of APDHE-Pareto should be interpreted primarily in terms of its explicit multi-objective, Pareto-based model-selection framework rather than as evidence of predictive superiority over conventional ensemble construction strategies. The ranking of the individual classifiers was largely preserved under the expanded search budget. ET, CatBoost, and GB remained the three highest-ranked individual classifiers by mean ROC-AUC, while LR and SVM remained the two lowest-ranked models; however, some changes occurred within the middle of the ranking, indicating that the ordering was not entirely insensitive to the search budget.

Following repeated nested cross-validation, the finalized model configurations were refitted using the complete development set and subsequently evaluated once on the held-out internal test set, which had remained isolated throughout model development. This evaluation provided a separate held-out internal assessment of the generalization performance of APDHE-Pareto and the individual candidate classifiers on previously unseen observations. The corresponding held-out test results are presented in Table 5.

When the APDHE-Pareto procedure was refitted on the complete development set, the final ensemble consisted of Gradient Boosting, LightGBM, and CatBoost, corresponding to an ensemble size of K = 3. The final decision threshold, selected from the fused training-only OOF probabilities using Youden’s J criterion, was 0.5400. This ensemble composition and threshold were fixed before evaluation on the held-out internal test set.

On the held-out internal test set, APDHE-Pareto achieved the highest ROC-AUC (0.9102), balanced accuracy (0.8281), F1-score (0.8136), and Matthews correlation coefficient (MCC; 0.6644), together with the lowest Brier score (0.1310) among the evaluated models. Among the individual classifiers, LightGBM achieved the highest ROC-AUC (0.9004), followed by LR (0.8945) and GB (0.8896). Although AdaBoost achieved the highest specificity (0.9375) and precision (0.9091), APDHE-Pareto demonstrated the strongest overall discriminative and probabilistic performance on the previously unseen held-out internal test set according to the evaluated performance measures. For complementary disease-oriented interpretation, the same frozen held-out predictions were additionally evaluated with gallstone presence defined as the positive class. Under this orientation, APDHE-Pareto achieved a sensitivity of 0.9063, specificity of 0.7500, precision of 0.7838, and F1-score of 0.8406 for gallstone-positive cases. For the prespecified ET comparator, the corresponding sensitivity, specificity, and precision were 0.8125, 0.7500, and 0.7647, respectively. These values represent a re-expression of the original held-out predictions and were obtained without model refitting, threshold re-optimization, or additional model selection.

To avoid post hoc comparator selection based on the held-out test results, the individual baseline was prespecified using the repeated nested cross-validation results obtained from the development set. ET was selected because it achieved the highest unrounded mean ROC-AUC among the individual classifiers during model development. Formal paired statistical comparisons between APDHE-Pareto and ET were subsequently performed using predictions generated for the same held-out test observations. The held-out ROC curves for APDHE-Pareto and ET are presented in Figure 2, with bootstrap-based 95% confidence intervals for the corresponding AUC estimates.

For formal statistical comparison, differences in paired classification outcomes were evaluated using the two-sided exact McNemar test, whereas differences in discriminative performance were assessed using the paired ROC-AUC difference estimated by stratified bootstrap resampling (5000 resamples) with a 95% confidence interval. The results of these paired statistical comparisons are summarized in Table 6.

In the prespecified statistical comparison performed on the held-out internal test set, APDHE-Pareto correctly classified five observations that were misclassified by ET, whereas ET correctly classified two observations that were misclassified by APDHE-Pareto. The two-sided exact McNemar test did not indicate a statistically significant difference in paired classification errors (*p* = 0.4531). APDHE-Pareto achieved a higher ROC-AUC than ET, with an estimated paired difference of 0.0420. However, the bootstrap-derived 95% confidence interval (−0.0068 to 0.0996) included zero, indicating that the observed difference was not statistically significant. Collectively, these findings indicate that, although APDHE-Pareto achieved numerically higher discriminative performance on the held-out internal test set, statistical superiority over the prespecified ET baseline was not demonstrated.

To further examine the contribution of the individual components of the proposed framework, an ablation analysis was performed by systematically modifying the information used during ensemble construction. Four configurations were evaluated: (i) Pareto-based performance selection alone, (ii) Pareto-based performance selection combined with prediction diversity, (iii) Pareto-based performance selection combined with cross-validation stability, and (iv) the complete APDHE-Pareto framework incorporating both prediction diversity and cross-validation stability. Ablation performance was assessed on unseen outer-validation observations to ensure that the comparisons reflected generalization performance rather than the out-of-fold predictions used during ensemble construction. The results of the ablation analysis are presented in Table 7.

The ablation analysis revealed only small differences among the four configurations. The Pareto-plus-stability configuration achieved the highest mean ROC-AUC (0.8601), balanced accuracy (0.7770), F1-score (0.7642), and MCC (0.5660), together with the lowest Brier score (0.1620). The full APDHE-Pareto configuration did not outperform this simpler variant, with corresponding values of 0.8585, 0.7755, 0.7629, 0.5623, and 0.1632, respectively. Adding prediction diversity to Pareto-based selection resulted in only minor changes in the mean performance measures, and the Pareto-plus-diversity and full APDHE-Pareto configurations yielded identical mean values. These results indicate that, in the present dataset and candidate-model pool, the incremental contribution of prediction diversity to predictive performance was limited, whereas the Pareto-plus-stability configuration showed the strongest numerical performance.

Across the 100 outer-validation evaluations, adding diversity to Pareto-only selection changed model ranking, ensemble composition, and ensemble size in 96%, 55%, and 41% of evaluations, respectively, whereas adding stability changed these outcomes in 90%, 48%, and 45%. When diversity was added after stability, the corresponding changes were 95%, 55%, and 41%; however, adding stability after diversity produced no further changes in ranking, composition, or ensemble size. These findings indicate that diversity and stability influenced the selection process, but their contributions were not uniformly additive. Accordingly, the ablation results do not indicate that every component was individually necessary to maximize predictive performance.

Separately, the ranking-sensitivity analysis showed that constituent selection was stable under two of the three alternative hierarchies examined. The original, stability-prioritized, and balanced-accuracy-prioritized hierarchies selected the same three-model ensemble (GB, LightGBM, and CatBoost; K = 3), with an OOF ROC-AUC of 0.8498. In contrast, the AUC-prioritized hierarchy selected GB and CatBoost (K = 2), with an OOF ROC-AUC of 0.8574. Thus, the selected ensemble was largely stable across the evaluated ranking schemes but was not invariant to the prespecified lexicographic ordering.

Following the ablation analysis, post hoc explainability analyses were performed to characterize the variables contributing to the predictions of the final APDHE-Pareto model. Figure 3 presents the global SHAP feature-attribution analysis together with an illustrative LIME explanation for the first observation in the held-out internal test set. These analyses were used to characterize global and local attribution patterns underlying model predictions and were interpreted exclusively as post hoc explanations of model behavior rather than as evidence of causal or etiological relationships between individual predictors and gallstone status.

Post hoc explainability analyses were performed to characterize the contribution of individual variables to APDHE-Pareto predictions. Global SHAP analysis identified C-reactive protein (CRP) as the variable with the highest mean absolute SHAP value, followed by vitamin D, bone mass, obesity, aspartate aminotransferase (AST), and the extracellular fluid-to-total body water ratio (ECF/TBW) (Figure 3a). CRP and vitamin D showed the largest global attribution magnitudes among the evaluated predictors, as reflected by their mean absolute SHAP values. Additional contributions were distributed across biochemical and body-composition variables, including visceral fat area, body protein content, visceral muscle area, lean mass, extracellular water, and low-density lipoprotein.

The local behavior of APDHE-Pareto was further examined using an illustrative LIME explanation for the first observation in the held-out internal test set (Figure 3b). For this observation, the model assigned a probability of 0.82 to the no-gallstone class and 0.18 to the gallstone class. CRP > 1.64 was the strongest positive contributor toward the no-gallstone prediction, followed by vitamin D ≤ 13.15. Bone mass ≤ 2.40 and AST ≤ 15.00 also supported the no-gallstone prediction, whereas ECF/TBW > 44.00 provided the strongest contribution in the opposite direction, toward the gallstone class. The prominence of CRP and vitamin D in the local explanation was consistent with their high global SHAP importance. Nevertheless, this LIME explanation represents the prediction behavior of the model for a single observation and should not be generalized to the overall study population. Accordingly, both SHAP and LIME results were interpreted as post hoc model-attribution measures rather than as evidence of causal or etiological relationships with gallstone status.

Calibration was additionally examined on the held-out internal test set using calibration curves, calibration intercept, and calibration slope (Figure 4). APDHE-Pareto yielded a calibration intercept of 0.168 and a calibration slope of 1.960, whereas the corresponding values for Extra Trees were 0.100 and 1.572, respectively. Thus, neither model showed ideal calibration, defined by an intercept of 0 and a slope of 1. Although APDHE-Pareto had a lower Brier score than Extra Trees (0.1310 vs. 0.1504), this difference was interpreted as reflecting overall probabilistic accuracy rather than calibration alone. Given the limited size of the held-out test set (*n* = 64), the calibration curves and intercept and slope estimates were interpreted descriptively.

## 4. Discussion

This study developed and evaluated APDHE-Pareto, an adaptive multi-objective ensemble framework for gallstone status classification using demographic, clinical, biochemical, anthropometric, and body-composition variables. The principal aim was to investigate whether complementary performance measures could be integrated with prediction diversity and cross-validation stability within a Pareto-based ensemble-selection framework, thereby avoiding predefined numerical weighting of the primary performance objectives. Across repeated nested cross-validation and evaluation on a held-out internal test set, APDHE-Pareto demonstrated competitive predictive performance while employing a systematic, leakage-controlled ensemble-selection strategy. These findings indicate that the principal contribution of APDHE-Pareto is a systematic and leakage-controlled framework for multi-objective ensemble selection while maintaining competitive predictive performance across multiple evaluation settings.

Across repeated nested cross-validation, APDHE-Pareto achieved the highest mean ROC-AUC while maintaining competitive performance across the remaining evaluation metrics. On the held-out internal test set, it also achieved the highest ROC-AUC, balanced accuracy, F1-score, MCC, and the lowest Brier score among the evaluated models. Several optimized individual classifiers also demonstrated strong predictive performance, highlighting the overall competitiveness of the candidate model pool. Collectively, these findings indicate that APDHE-Pareto provides a systematic and leakage-controlled multi-objective ensemble-selection framework capable of achieving competitive predictive performance across repeated cross-validation and held-out internal evaluation.

The additional comparison with stronger ensemble baselines provided a more direct assessment of whether the proposed selection framework offered an advantage over simpler ensemble construction strategies. Logistic stacking and uniform soft voting achieved modestly higher mean ROC-AUC values than APDHE-Pareto, whereas the remaining classification metrics were generally similar across the ensemble approaches. These results do not support a claim of predictive superiority for APDHE-Pareto over conventional ensemble methods in the present dataset. Rather, its distinction lies in the explicit integration of multi-objective performance assessment, prediction diversity, and stability within a leakage-controlled constituent-selection procedure.

The formal statistical comparison was restricted to Extra Trees, which was prospectively selected from the development results as the individual classifier with the highest unrounded mean ROC-AUC across the repeated nested cross-validation evaluations. This approach avoided retrospective comparator selection based on held-out test performance. APDHE-Pareto achieved a numerically higher ROC-AUC and correctly classified more held-out observations than Extra Trees. However, the paired statistical analyses did not identify a significant difference between the two approaches, likely reflecting the limited size of the held-out internal test set. Consequently, the numerical performance advantage observed for APDHE-Pareto should be interpreted within the context of the study’s statistical power and requires confirmation in larger independent cohorts.

The present findings are broadly consistent with previous studies demonstrating that gallstone status can be characterized using structured clinical, biochemical, and body-composition variables, as well as imaging-derived information. Previous ML investigations have identified variables such as CRP, vitamin D, body-composition measures, and liver-related biochemical markers as important contributors to gallstone classification. Consistent with these observations, the global SHAP analysis identified CRP as the variable with the highest mean absolute SHAP value, followed by vitamin D, bone mass, obesity, AST, and ECF/TBW. The illustrative LIME explanation for the selected held-out test observation likewise highlighted CRP and vitamin D as the strongest local contributors, consistent with their prominent importance in the global SHAP analysis. Nevertheless, SHAP and LIME quantify predictive attribution rather than causal or etiological effects. Accordingly, the identified variables should be interpreted as contributors to model predictions rather than as independent clinical risk factors for gallstone disease. Direct numerical comparison with previously published models should be interpreted cautiously because differences in study populations, predictor sets, preprocessing procedures, outcome definitions, and validation strategies may substantially influence reported performance estimates.

The ablation analysis provided additional insight into the relative contribution of the selection components. Performance differences among the evaluated configurations were small, with the Pareto-plus-stability configuration achieving the highest numerical performance. The full APDHE-Pareto framework did not provide an additional performance gain over this simpler configuration, and the incremental contribution of prediction diversity was limited in the present analysis. The additional selection-change analysis nevertheless showed that both diversity and stability frequently altered model ranking and ensemble construction across the outer-validation evaluations, although their effects were not uniformly additive. Therefore, the present results do not indicate that every component is individually necessary for maximizing predictive performance. These findings suggest that, for this dataset and candidate-model pool, cross-validation stability was associated with stronger numerical performance than prediction diversity. Accordingly, the practical role of the diversity component should be interpreted cautiously. Within APDHE-Pareto, diversity is intended to provide complementary information about redundancy and disagreement among candidate-model predictions rather than to ensure an improvement in predictive performance in every setting. Its contribution is therefore likely to depend on the extent to which the candidate models exhibit complementary prediction patterns. The present ablation results support a conditional rather than universal interpretation of the diversity component, and evaluation across larger datasets and broader candidate-model pools would be needed to determine the conditions under which diversity-based selection provides a measurable incremental benefit.

To place these findings in a broader methodological context, Table 8 compares APDHE-Pareto with selected recent ensemble-learning approaches. The comparison focuses on model-selection and optimization strategies, the treatment of diversity and stability, and ensemble fusion rather than on reported predictive performance, since the underlying datasets, prediction tasks, and validation procedures differ across studies.

In particular, Weng et al. [28] proposed a two-stage framework in which selected features and base-model probabilities are fused and provided to a CatBoost meta-learner, followed by threshold optimization. The two approaches differ mainly in how ensemble construction is organized. TSFFEM performs feature selection and probability-aware feature augmentation before meta-learning, whereas APDHE-Pareto applies Pareto non-dominated sorting directly to constituent-model selection without predefined weights for the primary performance metrics. Prediction diversity and cross-validation stability are then used as hierarchical selection characteristics, and the calibrated probabilities of the selected constituent models are combined through uniform averaging rather than through a separate meta-learner.

Several aspects of the study design strengthen the interpretation of these findings. Model development and final evaluation were strictly separated, with the held-out internal test set remaining isolated from hyperparameter optimization, probability calibration, constituent-model selection, ensemble construction, and decision-threshold optimization, thereby minimizing information leakage. Repeated nested cross-validation provided performance estimates across 100 outer validation evaluations, reducing dependence on a single data partition. Model performance was assessed using complementary discrimination, classification, and probabilistic metrics, while the formal statistical comparison employed a baseline selected exclusively from the development data. Ablation and post hoc explainability analyses further supported interpretation of both the ensemble-selection procedure and the resulting model predictions.

The findings of this study should be interpreted in light of several limitations. The analysis was based on a relatively small dataset comprising 319 observations obtained from a single source. The same publicly available dataset has also been used in several recent gallstone ML studies, including a recent stacking-based analysis, which further limits the extent to which the present findings can be considered independent validation evidence. Repeated nested cross-validation and a separate held-out internal test set provide complementary assessments of predictive performance, but they do not substitute for independent external validation. In addition, the repeated nested cross-validation design reduces information leakage and dependence on a single data partition but cannot eliminate model-selection instability arising from the combination of a relatively small sample and multiple data-dependent selection steps. The held-out test set contained only 64 observations, limiting statistical power and increasing uncertainty in model comparisons. The limited held-out sample size also increases uncertainty in the calibration curve and in the estimated calibration intercept and slope; these calibration results were therefore interpreted descriptively rather than as precise estimates of population-level calibration. Because both the development and held-out test sets originated from the same source dataset, the reported evaluation represents internal rather than external validation. In addition, some predictor information was derived from ultrasonographic assessment, and the source dataset does not provide sufficient information to establish the precise temporal availability of these ultrasound-derived predictors relative to the determination of gallstone status. Therefore, their availability before outcome determination cannot be assumed, and the present framework should not be interpreted as a pre-ultrasound screening or triage model. Prospective studies should ensure that all predictors are available at the intended point of prediction before evaluating clinical implementation. The findings may also be influenced by the specific predictor set, candidate classifier pool, hyperparameter search spaces, probability-calibration procedure, diversity measures, and stability definition adopted in the present framework. The constrained hyperparameter-search budget may also have limited the extent to which model-specific optima were explored, and relative model rankings may therefore exhibit some sensitivity to the search budget. A related methodological consideration concerns the lexicographic ranking applied after Pareto sorting. Although APDHE-Pareto avoids numerical scalarization of the four primary performance objectives at the Pareto stage, the subsequent ranking applies a prespecified hierarchy among Pareto layer, prediction diversity, cross-validation stability, and the remaining performance metrics. Accordingly, the overall selection procedure is not preference-free. The lexicographic hierarchy represents a methodological design choice rather than a uniquely optimal preference structure. The sensitivity analysis showed that ensemble selection remained unchanged under the stability- and balanced-accuracy-prioritized alternatives but changed when ROC-AUC was given earlier priority. Thus, constituent selection was largely stable across the evaluated ranking schemes, although it was not entirely insensitive to the prespecified preference order. Finally, this study evaluated predictive performance rather than clinical utility; no prospective evaluation, decision-curve analysis, or assessment of patient outcomes was performed. Consequently, the reported predictive performance should not be interpreted as evidence of clinical effectiveness without prospective external validation.

Future research should evaluate APDHE-Pareto in larger, multicenter, and independently collected cohorts to determine whether its predictive performance and ensemble composition remain stable across different populations and healthcare settings. External validation should assess both discrimination and probability calibration, with recalibration performed where appropriate. Future studies should also investigate alternative measures of prediction diversity and model stability, broader candidate-classifier pools, and comparisons with other Pareto-based multi-objective ensemble-selection frameworks. Prospective validation and decision-analytic studies will ultimately be required before considering clinical implementation. Such investigations will determine whether the methodological advantages of APDHE-Pareto translate into reproducible improvements beyond those achievable with optimized individual classifiers.

## 5. Conclusions

This study introduced APDHE-Pareto, an adaptive multi-objective ensemble framework for gallstone status classification that integrates multiple performance criteria with prediction diversity and cross-validation stability while avoiding numerical scalarization of the primary performance objectives during Pareto-based selection. In the primary model comparison, APDHE-Pareto showed competitive performance, including the highest mean ROC-AUC across repeated nested cross-validation and the highest ROC-AUC on the held-out internal test set. However, formal comparison with the prespecified ET baseline did not demonstrate statistically significant superiority. The ablation analysis further showed that the incremental contribution of prediction diversity was limited in the present dataset, while the Pareto-plus-stability configuration achieved the highest numerical performance among the ablation variants. These findings suggest that the contribution of individual selection components may depend on the dataset and candidate-model pool. Accordingly, the principal contribution of APDHE-Pareto lies in providing a systematic and leakage-controlled framework for multi-objective ensemble selection rather than in assuming that each additional component will necessarily improve predictive performance.

The present findings should be interpreted in light of the relatively small sample size and the absence of independent external validation. Although the proposed framework demonstrated encouraging performance, its generalizability and potential clinical utility remain to be established. Future studies should therefore evaluate APDHE-Pareto in larger, multicenter, and independently collected cohorts and compare it with alternative multi-objective ensemble-selection strategies.

## Figures and Tables

**Figure 1:**
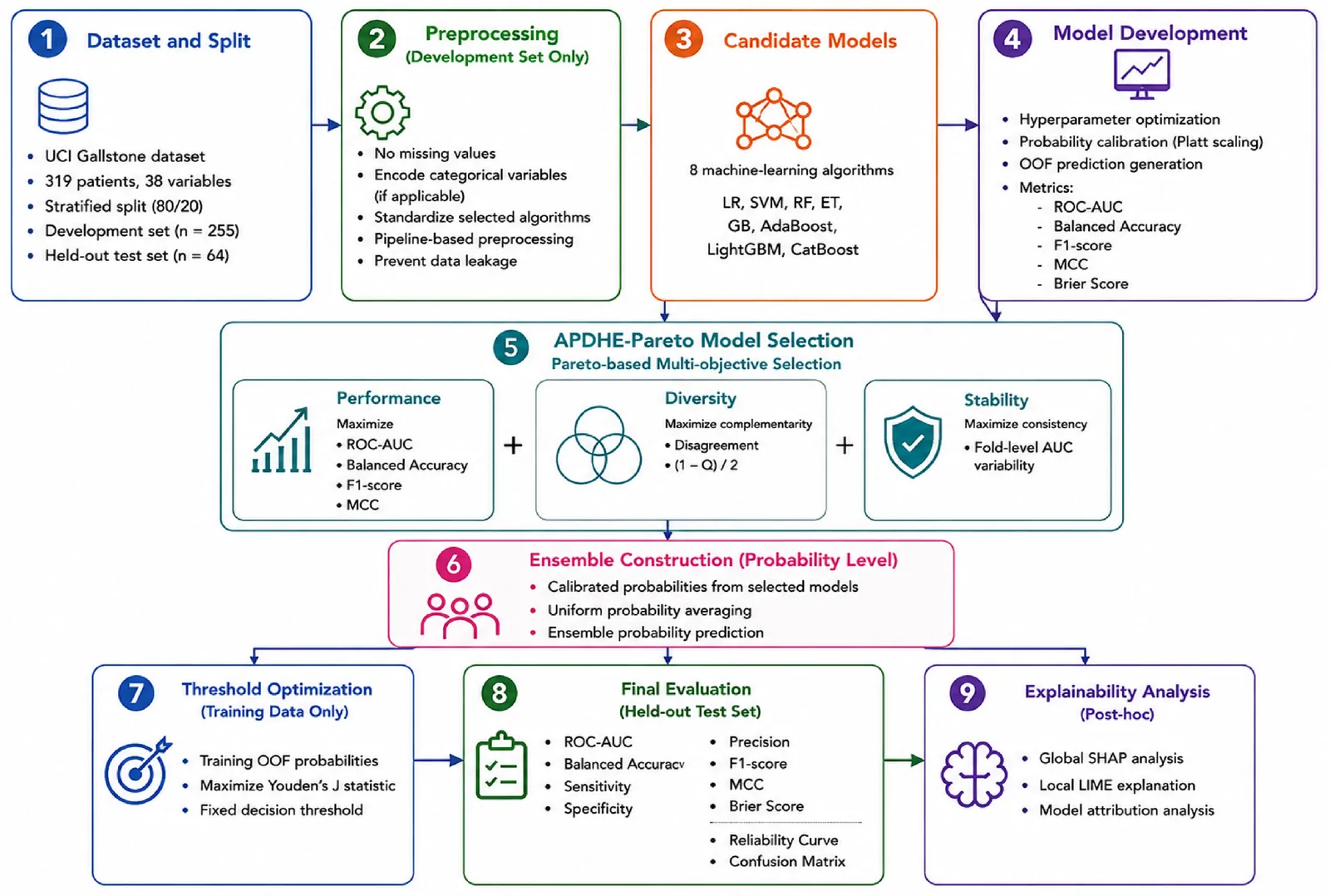
Study design and analytical workflow for gallstone status classification using the proposed APDHE-Pareto framework.

**Figure 2:**
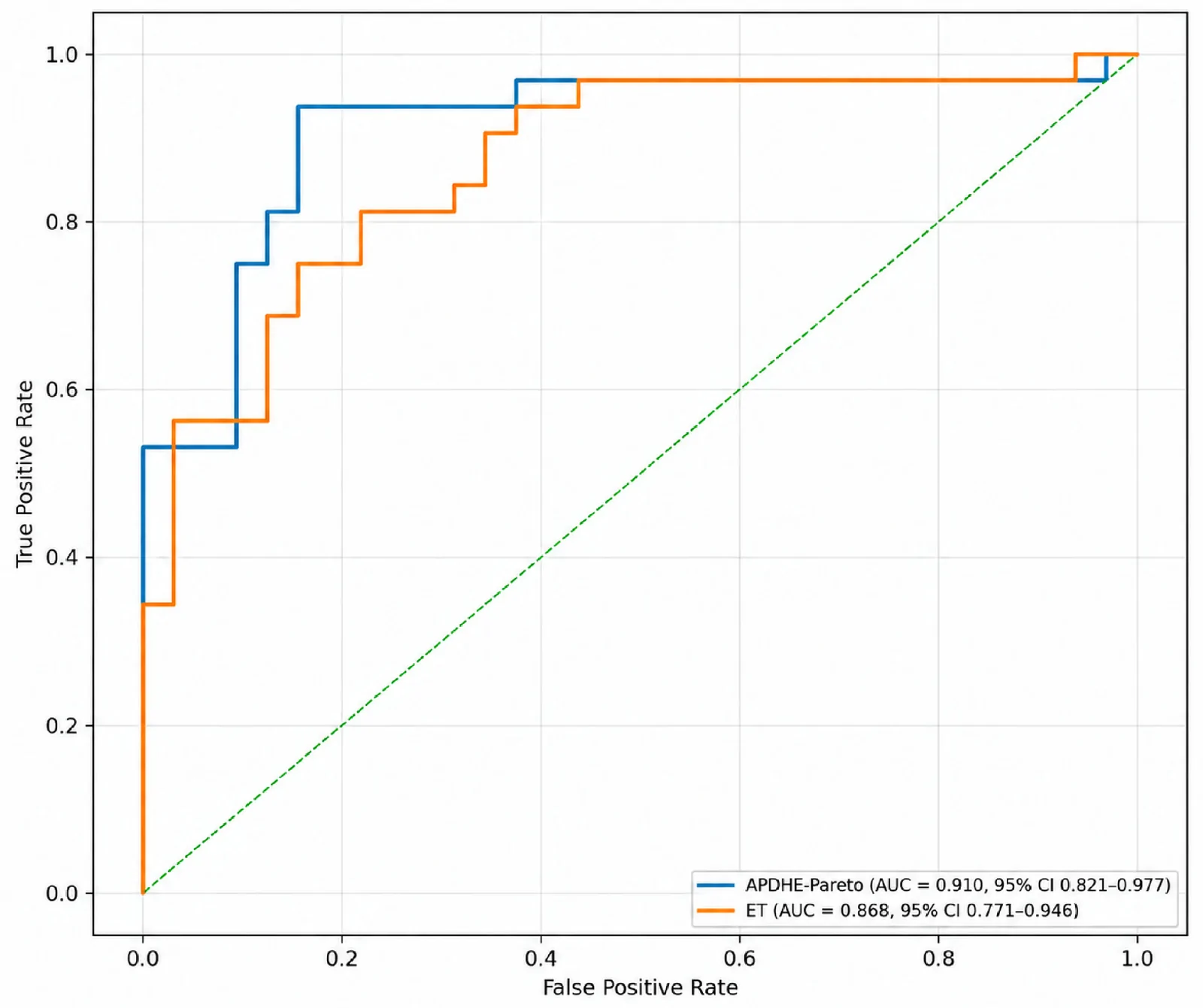
ROC curves of APDHE-Pareto and the prespecified ET baseline on the held-out internal test set, with bootstrap-based 95% confidence intervals for AUC.

**Figure 3:**
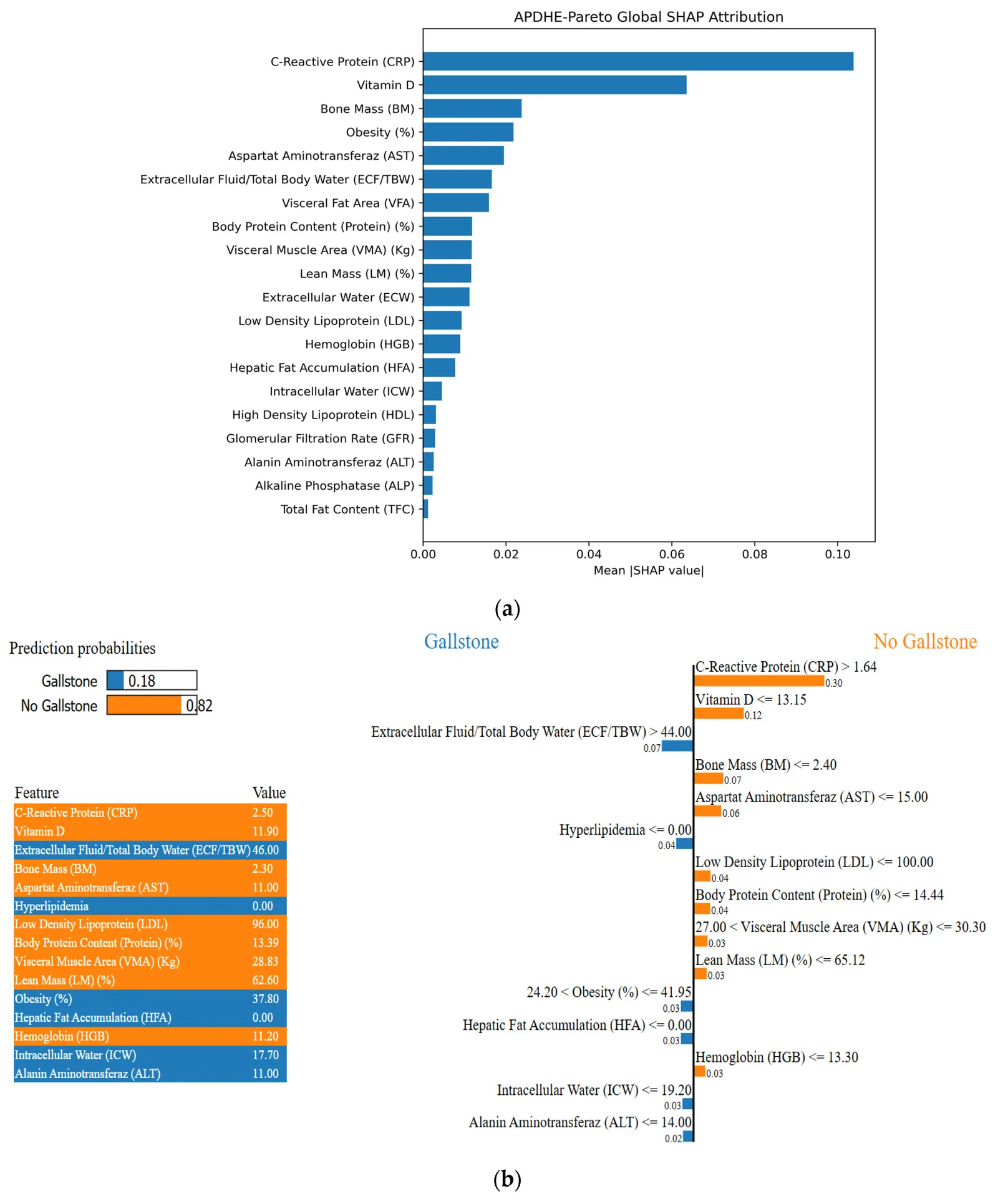
Global and local interpretation of APDHE-Pareto predictions. (**a**) Global feature importance quantified using mean absolute SHAP values. (**b**) LIME-based explanation for the first observation in the held-out internal test set, showing class probabilities and feature-specific contributions.

**Figure 4:**
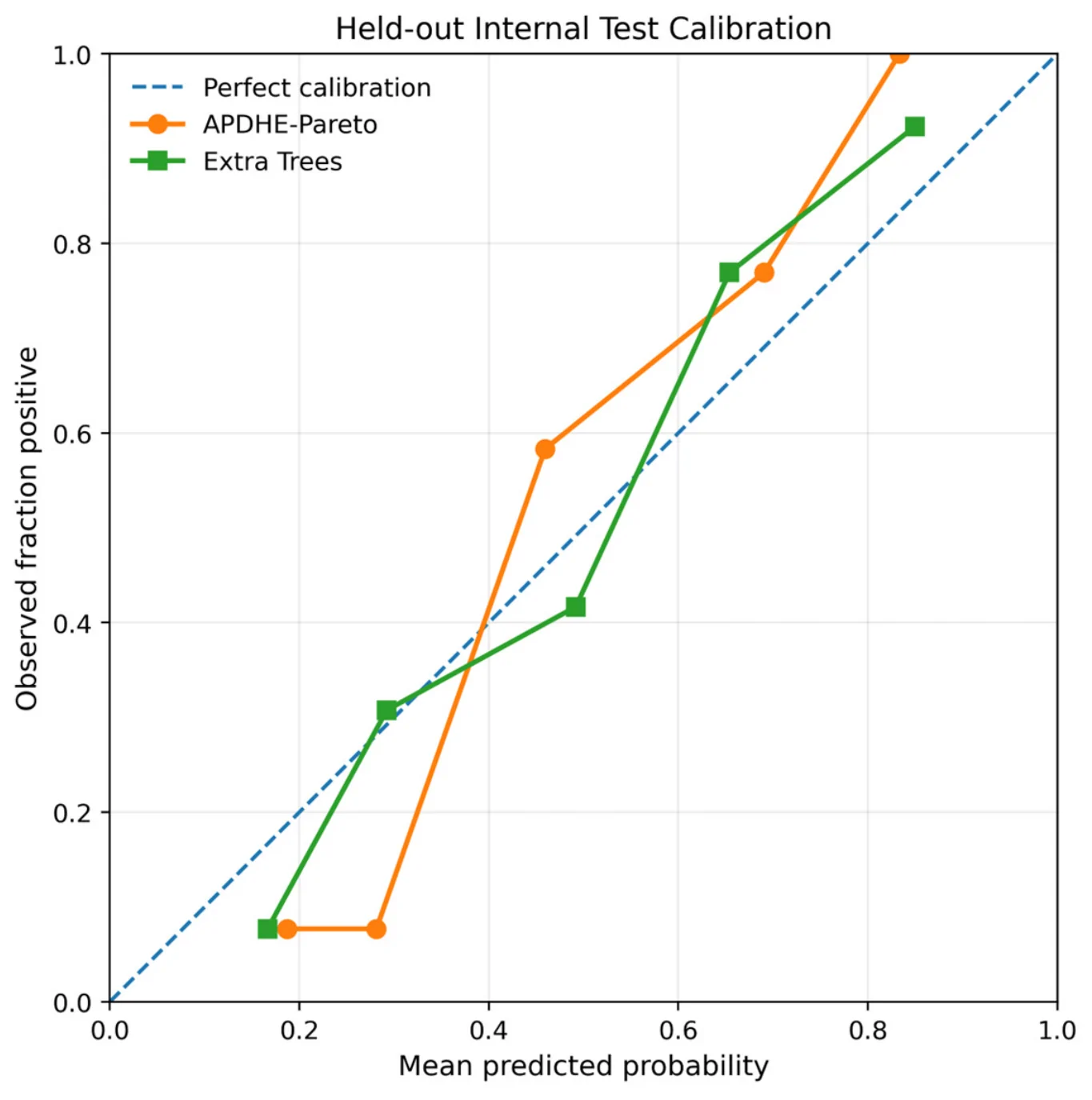
Calibration curves for APDHE-Pareto and the prespecified Extra Trees comparator on the held-out internal test set. Observed outcome frequencies are plotted against mean predicted probabilities across five quantile-based probability groups. The dashed diagonal line represents perfect calibration.

**Table 1:** Baseline characteristics of the study population according to gallstone status.

Variable	Overall (*n* = 319)	Gallstone (+) (*n* = 161)	Gallstone (−) (*n* = 158)
Age	49.00 [38.50–56.00]	49.00 [38.00–56.00]	50.00 [39.25–56.00]
BMI	28.30 [25.25–31.85]	27.70 [24.80–31.40]	29.15 [26.02–32.48]
Total Body Fat Ratio (%)	27.82 [22.02–34.81]	25.80 [20.10–31.70]	31.05 [23.99–36.02]
Visceral Fat Rating	9.00 [6.00–12.00]	9.00 [6.00–12.00]	9.00 [7.00–11.00]
Hepatic Fat Accumulation	1.00 [0.00–2.00]	1.00 [0.00–2.00]	2.00 [0.00–2.00]
Glucose	98.00 [92.00–109.00]	99.00 [93.00–110.00]	97.00 [91.00–107.75]
Total Cholesterol	198.00 [172.00–233.00]	199.00 [172.00–234.00]	198.00 [173.00–230.75]
LDL	122.00 [100.50–151.00]	125.00 [105.00–152.00]	119.00 [95.00–148.75]
HDL	46.50 [40.00–56.00]	45.00 [38.00–54.00]	47.50 [43.00–58.00]
Triglyceride	119.00 [83.00–172.00]	119.00 [82.00–188.00]	118.50 [89.00–163.75]
C-Reactive Protein	0.22 [0.00–1.62]	0.00 [0.00–0.31]	1.29 [0.20–3.45]
Vitamin D	22.00 [13.25–28.06]	25.27 [19.62–30.20]	17.00 [9.95–23.78]

**Table 2:** Hyperparameter search spaces for the candidate ML classifiers.

Algorithm	Hyperparameter	Search Space
LR	C	logspace(10^ − 2, 10^2), 9 values
	solver	lbfgs, liblinear
SVM	C	logspace(10^ − 2, 10^2), 9 values
	gamma	scale, auto
RF	n_estimators	200, 400, 600
	max_depth	None, 4, 6, 10
	min_samples_leaf	1, 2, 4
	max_features	sqrt, log2, 0.7
ET	n_estimators	200, 400, 600
	max_depth	None, 4, 6, 10
	min_samples_leaf	1, 2, 4
	max_features	sqrt, log2, 0.7
GB	n_estimators	100, 200, 300
	learning_rate	0.03, 0.05, 0.10
	max_depth	2, 3, 4
	subsample	0.8, 1.0
AdaBoost	n_estimators	100, 200, 300
	learning_rate	0.03, 0.05, 0.10, 0.5
	base-tree max_depth	1, 2, 3
LightGBM	n_estimators	200, 400, 600
	learning_rate	0.03, 0.05, 0.10
	num_leaves	15, 31, 63
	max_depth	−1, 4, 6, 10
	min_child_samples	10, 20, 30
CatBoost	iterations	200, 400, 600
	depth	3, 4, 6
	learning_rate	0.03, 0.05, 0.10
	l2_leaf_reg	1, 3, 5, 7

**Table 3:** Repeated nested cross-validation performance of the evaluated models.

Metric	APDHE-Pareto	ET	CatBoost	GB	LightGBM	RF	AdaBoost	LR	SVM
ROC-AUC	0.8585 ± 0.0818	0.8514 ± 0.0861	0.8496 ± 0.0771	0.8472 ± 0.0822	0.8396 ± 0.0798	0.8395 ± 0.0867	0.8322 ± 0.0764	0.8285 ± 0.0755	0.8087 ± 0.0922
Balanced Accuracy	0.7755 ± 0.0893	0.7717 ± 0.0910	0.7754 ± 0.0807	0.7732 ± 0.0808	0.7724 ± 0.0781	0.7634 ± 0.0861	0.7640 ± 0.0760	0.7584 ± 0.0850	0.7374 ± 0.0939
Sensitivity	0.7414 ± 0.1343	0.7562 ± 0.1182	0.7412 ± 0.1191	0.7431 ± 0.1098	0.7553 ± 0.1156	0.7474 ± 0.1198	0.7022 ± 0.1200	0.6853 ± 0.1183	0.7236 ± 0.1216
Specificity	0.8096 ± 0.1275	0.7872 ± 0.1300	0.8096 ± 0.1046	0.8033 ± 0.1114	0.7894 ± 0.1040	0.7794 ± 0.1150	0.8258 ± 0.1040	0.8314 ± 0.1102	0.7512 ± 0.1218
Precision	0.8027 ± 0.1132	0.7856 ± 0.1100	0.7978 ± 0.0947	0.7944 ± 0.1004	0.7836 ± 0.0904	0.7744 ± 0.1015	0.8061 ± 0.1014	0.8083 ± 0.1101	0.7457 ± 0.1047
F1-score	0.7629 ± 0.0994	0.7650 ± 0.0942	0.7631 ± 0.0893	0.7631 ± 0.0866	0.7642 ± 0.0851	0.7554 ± 0.0945	0.7436 ± 0.0897	0.7357 ± 0.0955	0.7297 ± 0.0983
MCC	0.5623 ± 0.1791	0.5508 ± 0.1827	0.5585 ± 0.1617	0.5539 ± 0.1626	0.5513 ± 0.1561	0.5332 ± 0.1727	0.5397 ± 0.1531	0.5294 ± 0.1720	0.4803 ± 0.1882
Brier Score	0.1632 ± 0.0312	0.1606 ± 0.0379	0.1592 ± 0.0370	0.1604 ± 0.0360	0.1663 ± 0.0343	0.1653 ± 0.0395	0.1708 ± 0.0278	0.1854 ± 0.0215	0.1836 ± 0.0327

Note: Values are presented as mean ± standard deviation across the 100 outer-validation evaluations. Because repeated cross-validation evaluations are statistically dependent, these values are reported descriptively and were not treated as independent observations for inferential confidence-interval estimation.

**Table 4:** Performance of strong ensemble baselines in the expanded-search sensitivity analysis across 100 repeated outer-validation evaluations.

Metric	Logistic Stacking	Uniform Soft Voting	Greedy Forward Ensemble	Top-k AUC Averaging	APDHE- Pareto
ROC-AUC	0.8689 ± 0.0748	0.8684 ± 0.0731	0.8651 ± 0.0765	0.8609 ± 0.0785	0.8613 ± 0.0792
Balanced accuracy	0.7772 ± 0.0893	0.7759 ± 0.0876	0.7753 ± 0.0819	0.7748 ± 0.0833	0.7763 ± 0.0838
Sensitivity	0.7386 ± 0.1288	0.7372 ± 0.1215	0.7571 ± 0.1171	0.7462 ± 0.1290	0.7437 ± 0.1299
Specificity	0.8159 ± 0.1133	0.8145 ± 0.1215	0.7934 ± 0.1295	0.8035 ± 0.1229	0.8089 ± 0.1128
F1-score	0.7635 ± 0.1017	0.7629 ± 0.0980	0.7678 ± 0.0884	0.7633 ± 0.0958	0.7637 ± 0.0962
MCC	0.5633 ± 0.1794	0.5611 ± 0.1769	0.5601 ± 0.1651	0.5596 ± 0.1659	0.5622 ± 0.1684
Brier score	0.1498 ± 0.0422	0.1583 ± 0.0289	0.1638 ± 0.0261	0.1623 ± 0.0300	0.1628 ± 0.0299

Note: Values are presented as mean ± standard deviation across 100 repeated outer-validation evaluations. The expanded-search sensitivity analysis used up to 20 randomized hyperparameter configurations per classifier. Standard deviations are descriptive because the repeated outer-validation evaluations are statistically dependent.

**Table 5:** Held-out internal test performance for gallstone status classification with bootstrap-based 95% CIs.

Model	ROC-AUC	Balanced Accuracy	Sensitivity	Specificity	Precision	F1-Score	MCC	Brier Score
LR	0.8945 (0.7949–0.9717)	0.8125 (0.7188–0.9063)	0.7188 (0.5625–0.8750)	0.9063 (0.7813–1.0000)	0.8846 (0.7692–1.0000)	0.7931 (0.6667–0.8966)	0.6363 (0.4454–0.8125)	0.1629 (0.1356–0.1938)
SVM	0.8535 (0.7470–0.9424)	0.7813 (0.6719–0.8750)	0.8125 (0.6563–0.9375)	0.7500 (0.5938–0.9063)	0.7647 (0.6579–0.8846)	0.7879 (0.6774–0.8824)	0.5636 (0.3480–0.7559)	0.1736 (0.1462–0.2029)
RF	0.8770 (0.7852–0.9551)	0.8125 (0.7188–0.9063)	0.7813 (0.6250–0.9063)	0.8438 (0.7188–0.9688)	0.8333 (0.7188–0.9583)	0.8065 (0.7000–0.9032)	0.6262 (0.4384–0.8141)	0.1420 (0.1016–0.1857)
ET	0.8682 (0.7715–0.9463)	0.7813 (0.6719–0.8750)	0.7500 (0.5938–0.9063)	0.8125 (0.6563–0.9375)	0.8000 (0.6857–0.9231)	0.7742 (0.6545–0.8750)	0.5636 (0.3480–0.7515)	0.1504 (0.1138–0.1918)
GB	0.8896 (0.7969–0.9619)	0.7969 (0.7031–0.8906)	0.7500 (0.5938–0.8750)	0.8438 (0.7188–0.9688)	0.8276 (0.7143–0.9583)	0.7869 (0.6667–0.8889)	0.5964 (0.4064–0.7847)	0.1423 (0.1017–0.1879)
AdaBoost	0.8779 (0.7793–0.9561)	0.7813 (0.6875–0.8750)	0.6250 (0.4688–0.7813)	0.9375 (0.8438–1.0000)	0.9091 (0.7895–1.0000)	0.7407 (0.5882–0.8621)	0.5922 (0.4045–0.7635)	0.1500 (0.1138–0.1896)
LightGBM	0.9004 (0.8086–0.9678)	0.8125 (0.7188–0.9063)	0.7500 (0.5938–0.8750)	0.8750 (0.7500–0.9688)	0.8571 (0.7419–0.9643)	0.8000 (0.6786–0.9000)	0.6299 (0.4384–0.8141)	0.1491 (0.1206–0.1818)
CatBoost	0.8877 (0.7988–0.9600)	0.8125 (0.7188–0.9063)	0.7813 (0.6250–0.9063)	0.8438 (0.7188–0.9688)	0.8333 (0.7188–0.9565)	0.8065 (0.6909–0.9032)	0.6262 (0.4375–0.8125)	0.1452 (0.1080–0.1862)
APDHE-Pareto	0.9102 (0.8213–0.9766)	0.8281 (0.7344–0.9063)	0.7500 (0.5938–0.9063)	0.9063 (0.7813–1.0000)	0.8889 (0.7778–1.0000)	0.8136 (0.6923–0.9063)	0.6644 (0.4745–0.8272)	0.1310 (0.0970–0.1702)

Note: Values are presented as point estimates with bootstrap-based 95% CIs in parentheses. CIs were estimated using 5000 stratified percentile bootstrap resamples of the held-out internal test set. In the primary analysis, gallstone absence was treated as the positive class; therefore, sensitivity and precision refer to the identification of individuals without gallstones, whereas specificity refers to the identification of gallstone cases.

**Table 6:** Statistical comparison of APDHE-Pareto and the prespecified ET baseline on the held-out internal test set.

Comparison	APDHE Correct/ ET Wrong	APDHE Wrong/ ET Correct	McNemar *p*-Value	ΔAUC	95% CI
APDHE-Pareto vs. ET	5	2	0.4531	0.0420	−0.0068 to 0.0996

**Table 7:** Ablation analysis of the APDHE-Pareto framework across repeated outer-validation folds.

Configuration	ROC-AUC	Balanced Accuracy	F1-Score	MCC	Brier Score
Pareto performance only	0.8587	0.7732	0.7607	0.5578	0.1629
Pareto + diversity	0.8585	0.7755	0.7629	0.5623	0.1632
Pareto + stability	0.8601	0.7770	0.7642	0.5660	0.1620
Full APDHE-Pareto	0.8585	0.7755	0.7629	0.5623	0.1632

**Table 8:** Methodological comparison of APDHE-Pareto with selected recent ensemble-learning frameworks.

Study/Framework	Primary Selection or Optimization Strategy	Diversity/Stability	Fusion Strategy	Main Distinction from APDHE-Pareto
Li et al. [26]	PCA-based dimensionality reduction combined with MI-, L1-, and RF-based feature selection	Diversity and cross-validation stability are not reported as explicit constituent-selection criteria	RF, XGBoost, and SVM outputs are combined through an LR meta-classifier	Uses a predefined stacking architecture after feature extraction and selection rather than Pareto-based adaptive constituent selection
Zhou et al. [30]	Multi-objective feature selection is used to generate accurate and diverse individual classifiers	Diversity is assessed through a feature-relevance-guided criterion during ensemble selection	Ensemble selection is performed among classifiers generated from different feature subsets	Multi-objective optimization is applied primarily to feature-subset generation rather than directly to heterogeneous candidate-model selection
Mohtasham et al. [29]	Hybrid feature selection followed by diversity-guided stacking	Explicit diversity measures (Disagreement, Yule’s Q, Cohen’s Kappa, Entropy, Kohavi-Wolpert) are used to construct candidate sub-model sets	Selected base learners are combined through a stacking meta-learner	Diversity directly guides stacking configuration, whereas APDHE-Pareto first performs multi-metric Pareto ranking and subsequently uses diversity and stability hierarchically
Moradpour et al. [31]	NSGA-II-based multi-objective hyperparameter optimization across multiple classification criteria	Variability across performance measures is incorporated through a standard-deviation objective	Pareto-derived solutions are incorporated into an AdaBoost-based ensemble	Pareto optimization targets classifier configurations and hyperparameter criteria rather than direct constituent selection from heterogeneous candidate models
Weng et al. [28]	Tree-based feature selection followed by probability-aware feature augmentation	Diversity and cross-validation stability are not used as hierarchical constituent-selection criteria	Selected features and base-model probabilities are integrated through a CatBoost meta-learner	Uses probability-aware feature augmentation and meta-learning rather than Pareto-based constituent-model selection followed by uniform probability averaging
APDHE-Pareto	Pareto non-dominated sorting of heterogeneous candidate classifiers using ROC-AUC, balanced accuracy, F1-score, and MCC	Prediction diversity and cross-validation stability are used hierarchically after Pareto performance ranking	Uniform averaging of calibrated probabilities from adaptively selected constituents	Applies multi-objective optimization directly to constituent-model selection, with ensemble size and decision threshold determined from training-only OOF predictions

## Data Availability

The dataset analyzed in this study is publicly available from the UCI Machine Learning Repository under the Gallstone dataset and can be accessed at UCI Machine Learning Repository—Gallstone Dataset (https://archive.ics.uci.edu/dataset/1150/gallstone-1, accessed on 18 September 2026). The source code developed for the APDHE-Pareto framework and the corresponding analysis scripts are available from the corresponding author upon reasonable request.
